# Genetic analyses of bone morphogenetic protein 2, 4 and 7 in congenital combined pituitary hormone deficiency

**DOI:** 10.1186/1472-6823-13-56

**Published:** 2013-12-01

**Authors:** Jana Breitfeld, Susanne Martens, Jürgen Klammt, Marina Schlicke, Roland Pfäffle, Kerstin Krause, Kerstin Weidle, Dorit Schleinitz, Michael Stumvoll, Dagmar Führer, Peter Kovacs, Anke Tönjes

**Affiliations:** 1 Department of Medicine, University of Leipzig, Liebigstrasse 20, Leipzig 04103, Germany; 2 IFB Adiposity Diseases, University of Leipzig, Philipp-Rosenthal-Str. 27, Leipzig 04103, Germany; 3 Hospital for Children & Adolescents, University of Leipzig, Liebigstrasse 22, Leipzig 04103, Germany; 4 Department of Endocrinology, University of Essen, Hufelandstraße 55, Essen 45147, Germany

**Keywords:** Combined pituitary hormone deficiency, Bone morphogenetic proteins, BMP2, BMP4, BMP7

## Abstract

**Background:**

The complex process of development of the pituitary gland is regulated by a number of signalling molecules and transcription factors. Mutations in these factors have been identified in rare cases of congenital hypopituitarism but for most subjects with combined pituitary hormone deficiency (CPHD) genetic causes are unknown. Bone morphogenetic proteins (BMPs) affect induction and growth of the pituitary primordium and thus represent plausible candidates for mutational screening of patients with CPHD.

**Methods:**

We sequenced *BMP2*, *4* and *7* in 19 subjects with CPHD. For validation purposes, novel genetic variants were genotyped in 1046 healthy subjects. Additionally, potential functional relevance for most promising variants has been assessed by phylogenetic analyses and prediction of effects on protein structure.

**Results:**

Sequencing revealed two novel variants and confirmed 30 previously known polymorphisms and mutations in *BMP2*, *4* and *7*. Although phylogenetic analyses indicated that these variants map within strongly conserved gene regions, there was no direct support for their impact on protein structure when applying predictive bioinformatics tools.

**Conclusions:**

A mutation in the *BMP4* coding region resulting in an amino acid exchange (p.Arg300Pro) appeared most interesting among the identified variants. Further functional analyses are required to ultimately map the relevance of these novel variants in CPHD.

## Background

The development of the pituitary gland is a highly complex process, involving many signalling molecules and transcription factors [1-3]. During embryogenesis cells from the oral ectoderm form the adenohypophysis, while the posterior part develops from neural tissue. With the help of animal models it has been shown that transcription factors like HesX1, Prop1, Pou1F1, Lhx3, Lhx4, Pitx1, Pitx2, Otx2, Sox2 and Sox3 play a crucial role in the development of the pituitary gland [4-6]. Several mutations in genes encoding these transcription factors have been reported in combined pituitary hormone deficiency (CPHD). However, for most of the patients the genetic cause of hypoplasia or at least functional insufficiency of the pituitary gland remains to be discovered.

Bone morphogenetic proteins (BMP) 2, 4 and 7 have a crucial role during the embryonic development of the pituitary gland [7]. In early development Bmp4 contributes to the formation of the rudimentary Rathke’s pouch in the mouse (reviewed in [4]). Later BMP 2, 4 and 7 secreted by surrounding tissues contribute to the polarisation of the pouch [7,8]. The development of the pituitary gland is completed within the first trimester of pregnancy in humans [9].

The BMPs are members of the transforming growth factor (TGF)-ß family and bind to type 1 and 2 serine-threonine kinase receptors (BMPR1A and BMPR2). Among different isoforms, three type 1 receptors (BMPR1A/ALK3, BMPR1B/ALK6, and ACVR1A/ALK2) and three type 2 receptors (BMPR2, ACTR2A, and ACTR2B) mediate most of the effects of BMPs [10-14]. *Bmp2* null mice die between embryonic day E.7.5 and E.10.5, suffering from cardiac defects [15]. Selective inhibition of *Bmp4* in mouse embryos results in a loss of nearly all pituitary cell lines except a few corticotrophs [8]. *Bmp4* knock-out mice are characterized by pituitary aplasia, suffer from severe facial, kidney and skeletal abnormalities, and die early in embryogenesis [16]. Severe eye defects and skeletal and renal anomalies are found also in *Bmp7* null mice [17], which die shortly after birth [18]. However, systematic search for mutations in *BMP2, 4* and *7* in patients with combined pituitary insufficiency has not been performed yet. So, the aim of our study was to investigate whether genetic variants in *BMP2*, *BMP4* and/or *BMP7* are associated with congenital pituitary insufficiency.

## Methods

### Subjects

In the present study, we included 19 patients (13 males, 6 females) with congenital combined pituitary hormone insufficiency (Table 1). Prior to direct sequencing of *BMP* genes, screening for known mutations in PIT1 and PROP1 has been performed in 19 subjects and did not reveal any aberrant results. Screenings for mutations in further genes are specified in Table 1.

**Table 1:** Patient characteristics

**Pat**	**Sex**	**Genetic screening**	**Further genetic tests**	**Lack of**				**Pituitary gland in MRI-scan**	**Special aspects**	**Symptoms leading to diagnosis**	**Age of diagnosis**	**Family history**
				**GH**	**TSH**	**LH/FSH**	**ACTH**					
**1**	m	PIT1, PROP1, HESX1, LHX3	/	+	+	+	+	hypoplastic	/	growth retardation and hypopituitarism	childhood/adolesence	no
**2**	m	PIT1, PROP1, HESX1, LHX3	SOX2, OTX2	+	+	+	+	hypoplastic	midline defect, right anophthalmia, mental retardation	severe malformations	birth	no
**3**	f	PIT1, PROP1, HESX1, LHX3	/	+	+	+	+	hypoplastic	brain atrophy	growth retardation	childhood/adolesence	no
**4**	f	PIT1, PROP1, HESX-1	/	+	+	no	no	hypoplastic	/	growth retardation	childhood/adolesence	no
**5**	m	PIT1, PROP1, HESX-1, LHX3	LHX4	+	+	+	+	hypoplastic	sclerosed nodules at the hands, short metacarpalia IV, azoospermia,	growth retardation	childhood/adolesence	no
**6**	m	PIT1, PROP1, HESX1, LHX3	LHX4	+	+	no	+	n.a.	/	unknown		
**7**	f	PIT1, PROP1, HESX1, LHX3	/	+	+	no	+	small and ectopic neuropituitary gland	left optic atrophy	growth retardation, postpartal hypoglycaemia	childhood/adolesence	no
**8**	m	PIT1, PROP1, HESX1, LHX3	GLI2	+	+	no	+	small pituitary and ectopic neuropituitary gland	/	unknown	unknown	no
**9**	m	PIT1, PROP1, HESX1, LHX3	/	no	+	no	+	ectopic adeno- and neuropituitary gland	/	prolonged jaundice, hypothyroidism	early infancy	no
**10**	f	PIT1, PROP1, HESX1, LHX3	/	+	+	+	+	hypoplastic	/	hypoglycaemia, hypothyroidism	early infancy	no
**11**	m	PIT1, PROP1	LHX4, GLI2	+	no	+	+	n.a.	Asperger syndrome	unknown		
**12**	m	PIT1, PROP1, HESX1, LHX3	/	+	+	+	+	n.a.	/	growth retardation, puperty onset at the age of 18, hypogonadism	childhood/adolesence	yes
**13**	m	PIT1, PROP1, HESX1, LHX3	/	+	+	no	+	n.a.	/	unknown		
**14**	m	PIT1, PROP1, HESX1, LHX3	/	+	+	+	+	hypoplastic	/	pericardial effusion	adulthood	yes
**15**	m	PIT1, PROP1, HESX1, LHX3	/	+	+	no	+	normal size, but ectopic neuropituitary gland	/	prolonged jaundice, hypoglycaemia, micropenis, muscular hypotonia, hypothyroidism	early infancy	no
**16**	f	PIT1, PROP1, HESX1, LHX3	LHX4	+	+	+	+	hypoplastic	/	hypoglycaemia, hyponatraemia, hepatopathy, muscular hypotonia	3rd day of life	n.a.
**17**	m	PIT1, PROP1, HESX1, LHX3	GLI2, SHH	+	+	+	+	hypoplastic	/	complex facial malformations	childhood/adolesence	no
**18**	m	PIT1, PROP1, HESX1, LHX3	/	+	+	+	+	small and ectopic neuropituitary gland	arachnodactyly, pulmonalisectasia, cryptorchidism, scoliosis	unknown		
**19**	f	PIT1, PROP1, HESX1, LHX3	/	+	+	no	+	hypoplastic	/	growth retardation	childhood/adolesence	no

m = male; f = female; MRI-scan = magnetic resonance imaging; MPHD = screening for PIT1, PROP1, HESX1, LHX3; PIT1 = POU domain, class 1, transcription factor 1; PROP1 = Homeobox protein prophet of Pit-1; HesX-1 = HESX homeobox 1; SOX2 = SRY (sex determining region Y)-box 2; OTX2 = SRY (sex determining region Y)-box 2 ; LHX4 = LIM/homeobox protein Lhx4; GLI2 = Zinc finger protein GLI2; SHH = Sonic hedgehog homolog; GH = growth hormone; TSH = thyroid stimulating hormone; LH = luteinizing hormone; FSH = follicle stimulating hormone; ACTH = adrenocorticotropic hormone; n.a. = not available.

To determine the frequency of newly identified genetic variants in the general population, we included a set of 1046 healthy subjects (Germany) without any history of pituitary disorders [19].

The study was approved by the ethics committee of the University of Leipzig and all subjects provided written informed consent before taking part in the study.

### DNA extraction and sequencing

Genomic DNA was extracted from lymphocytes using the Fujifilm (Düsseldorf, Germany) QuickGene DNA whole blood kit according to the manufacturer’s protocol. We sequenced all exons, exon-intron boundaries, 5′- and 3′-untranslated regions (UTR) of *BMP2* (Ensembl ENSG00000125845)*, BMP4* (Ensembl ENSG00000125378) and *BMP7* (Ensembl ENSG00000101144) in DNA samples from 19 non-related Caucasian subjects. Sequencing was performed using the Big Dye® Terminator (Applied Biosystems, Inc., Foster City, CA) on an automated DNA capillary sequencer (ABI PRISM® 3100 Avant; Applied Biosystems, Inc., Foster City, CA). Sequence information and PCR conditions for all oligonucleotide primers used for variant screening are available in Additional files 1 and 2. Known single nucleotide polymorphisms (SNPs) are designated according to dbSNP (http://www.ncbi.nlm.nih.gov/snp/) reference accession numbers.

### Prediction of functional relevance

To predict the potential impact of an identified variant on protein structure and function we used several online tools and databases: SIFT (http://sift.bii.a-star.edu.sg/) [20], PolyPhen (http://genetics.bwh.harvard.edu/pph/) [21], Mutpred (http://mutpred.mutdb.org) [22], FATHMM (http://fathmm.biocompute.org.uk) [23], Mutation Taster (http://www.mutationtaster.org) [24], SNP and Go (http://snps-and-go.biocomp.unibo.it/snps-and-go) [25].

### Genotyping of novel variants in control subjects

We genotyped all newly identified variants that predict an amino acid exchange in the cohort of healthy subjects by employing the TaqMan allelic discrimination assay (Applied Biosystems, Inc., Foster City, CA). The genotypes were detected on an ABI PRISM 7500 sequence detector (Applied Biosystems Inc.) according to the manufacturer’s protocol. Genotyping success rates for all analyzed SNPs were 99%.

### Phylogenetic analysis of the newly identified *BMP4* variant c.899G > C

For the coding region of *BMP4*, the conservation between species was determined by using Phylogenetic Analysis by Maximum Likelihood (PAML) [26]. Specifically, the aim of this analysis was to identify the ratio of non-synonymous to synonymous base substitutions (omega, ω=dN/dS). The coding sequences of 37 *BMP4* orthologues were downloaded from ENSEMBL (http://www.ensembl.org) and the NCBI (http://www.ncbi.nlm.nih.gov) databases.

## Results

### BMP2

Direct sequencing of *BMP2* revealed 10 that have been previously reported. The non-synonymous SNP rs2273073, found to be heterozygous in one out of 19 analyzed subjects represents a T to G base pair exchange resulting in a serine to alanine amino acid (aa) substitution at protein position 37 (p.Ser37Ala). A second non-synonymous variant (rs235768) also located within the coding region results in an arginine to serine exchange (p.Arg190Ser) and was found with a minor allele frequency (MAF) of 0.34 in our analyzed cohort. Detailed information of all identified SNPs in *BMP2* is presented in Table 2.

**Table 2:** **SNPs within *BMP2/4/7 *** **identified by sequencing of 19 subjects with congenital combined pituitary hormone insufficiency**

**Gene region**	**Exon/Intron**	**SNP**	**MAF according to NCBI**	**MM/mm in analyzed cohort**	**aa-exchange**	**MAF in analyzed cohort**
*BMP2* (ENST00000378827, NM_001200.2)						
5′-UTR	Exon 1^#^	rs35123420	C = 0.040	G/C		C = 0.026
	Exon 1	rs141364472	n.a.	G/A		A = 0.026
	Exon 2	rs2273073	G = 0.028	T/G	p.Ser37Ala	G = 0.026
coding	Exon 2	rs1049007	A = 0.250	G/A	synonymous	A = 0.342
region	Exon 3	rs235768	A = 0.240	T/A	p.Arg190Ser	A = 0.342
	Exon 3	rs13037675	T = 0.046	C/T	synonymous	T = 0.026
	Exon 3	rs15705	C = 0.280	A/C		C = 0.368
3′-UTR	Exon 3	rs3178250	C = 0.264	T/C		C = 0.368
	Exon 3	rs235769	A = 0.234	G/A		A = 0.368
	Exon 3	rs170986	A = 0.162	C/A		A = 0.053
*BMP4* (ENST00000245451, NM_001202.3)						
5′-UTR	Intron 2	rs2855532	T = 0.427	C/T		T = 0.342
	Intron 2	rs2761880	T = 0.221	C/T		T = 0.053
coding	Exon 4	rs17563	C = 0.373	C/T	p.Val152Ala	T = 0.447
region	**Exon 4**	**c.899G > C**		**G/C**	**p.Arg300Pro**	**C = 0.026**
*BMP7* (ENST00000395863, NM_001719.2)						
	Exon 2	rs41274738	T = 0.018	C/T	synonymous	T = 0.026
	Intron 2*	rs192121279	n.a.	G/A	p.Thr105Met	A = 0.026
	Intron 2*	rs6070031	T = 0.281	C/T		T = 0.421
	**Intron 2**	**c.611 + 3366C > T**		**C/T**		**T = 0.026**
coding	Exon 4	rs61733436	T = 0.005	C/T	synonymous	T = 0.026
region	Intron 4	rs6014948	T = 0.069	C/T		T = 0.053
	Intron 4	rs6070008	T = 0.466	A/T		T = 0.421
	Exon 5	rs61733438	C = 0.005	T/C	p.Asn321Ser	C = 0.026
	Intron 6^$^	rs2148328	A = 0.466	A/G	p.Ala399Gly	G = 0.474
	Intron 7	rs10375	C = 0.484	C/T		T = 0.447
	Intron 7	rs151255710	n.a.	A/G		G = 0.026
	Intron 7	rs17480735	A = 0.051	G/A		A = 0.105
	Intron 7	rs6025418	G = 0.479	A/G		G = 0.447
3′-UTR	Intron 7	rs6025417	C = 0.478	G/C		C = 0.447
	Intron 7	rs6025416	C = 0.452	T/C		C = 0.447
	Intron 7	rs6014947	T = 0.460	C/T		T = 0.474
	Intron 7	rs6025415	C = 0.478	G/C		C = 0.473
	Intron 7	rs6014946	C = 0.461	A/C		C = 0.473

SNP = single nucleotide polymorphism, BMP = bone morphogenetic protein; MAF = minor allele frequency; MM = major allele, mm = minor allele in analyzed cohort; aa = amino acid; UTR = untranslated region; n.a. = not available; novel identified SNPs are presented in bold; ^#^) only in ENST00000378827 but not in NM_001200 part of exon 1 (5′UTR) ; *) variants are located within an additional exon only present in isoform BMP7 ENST00000433911; ^$^) for transcript variant ENST00000450594 the SNPs is located within the coding region.

### BMP4

Sequencing of the *BMP4* gene revealed four SNPs. Three of them have already been described by others (Table 2). The newly identified variant c.899G > C leads to an aa exchange from arginine to proline at position 300 (p.Arg300Pro) within the protein and was found as a heterozygous mutation in one of the 19 analyzed subjects (patient number 5; Table 1). Genotyping of this variant in 1046 healthy subjects did not reveal any further heterozygous or homozygous c.899G > C carrier. Additionally, we have found the non-synonymous variant rs17563, resulting in a p.Val152Ala substitution. This SNP was found with a MAF of 0.45 within the cohort. PAML analyses showed an overall strong conservation of the gene. Positional analyses further indicated that most positions are conserved or strongly conserved. Position number 300 is highly conserved. Regarding each species separately revealed that the human lineage seems to have no synonymous substitutions leading to an infinite omega. The absence of synonymous changes in the data leads to the infinite omega, as there is a positive number divided by zero. A likelihood ratio test (LRT) against the model with the average omega reveals that P < 0.005, underlining that we have a strongly conserved gene.

All identified genetic variants in the *BMP4* gene are presented in Table 2.

### BMP7

All protein-coding exons that constitute the various transcripts of *BMP7* were sequenced. In total we found 18 genetic variants. One variant (c.611 + 3366C > T) has not been reported so far. The novel intronic SNP at position c.611 + 3366C > T showed a MAF of 0.026. This variation was found in patient number 17 (Table 1). This subject presented a phenotype including a hypoplastic pituitary gland and complex facial malformations. The previously known genetic variant rs61733438 results in the aa exchange p.Asn321Ser and was found in one out of the 19 subjects. Finally, two further previously known SNPs (rs192121279 and rs2148328) resulted in the aa substitutions p.Thr105Met and p.Ala399Gly each in one of the known BMP7 isoforms (ENST00000433911 and ENST00000450594). The results for sequencing of *BMP7* are summarized in Table 2.

#### Prediction of functional relevance

Potential impacts of all newly identified variants on protein structure and function was investigated by use of several online tools [20-25]. The only variant with consistent evidence for functional consequences was c.899G > C in *BMP4*. Results are summarized in Table 3.

**Table 3:** Assessment of potential functional relevance of identified variants

		**Sift **[20]	**PolyPhen **[21]	**Mutation taster **[22]	**SNP&GO **[23]	**FATHMM **[24]	**MutPred **[25]
**BMP2**							
rs2273073	Ser37Ala	- tolerated	- tolerated	- aas changed	- aas changed	- tolerated	- score 0.123
				- heterozygous in TGP		- score 0.61	- neutral
				- known disease mutation at this position (HGMD CM034611)	- disease associated variation		
				- protein features (might be) affected	(probability – 0.527)		
				- splice site changes			
rs235768	Arg190Ser	- functional relevance cannot be predicted explicit	- functional relevance cannot be predicted explicit	- aas changed	- aas changed	- tolerated	- score 0.293
				- homozygous in TGP		- score -0.45	- neutral
				- protein features (might be) affected	- disease associated variation		
				- splice site changes	(probability – 0.974)		
**BMP4**							
rs17563	Val152Ala	- tolerated	- tolerated	- aas changed	- aas changed	- tolerated	- score 0.145
				- homozygous in TGP		- score -0.08	- neutral
				- protein features (might be) affected	- disease associated variation		
				- splice site changes	(probability – 0.755)		
c.899G > C	Arg300Pro	- substantial evidence for functional consequences	- substantial evidence for functional consequences	- disease causing	- aas changed	- tolerated	- score 0.381
				- aas changed		- score -0.78	- neutral
				- protein features (might be) affected	- disease associated variation		
				- splice site changes	(probability – 0.906)		
**BMP7**							
rs192121279	Thr105Met	- deleterious	- unknown	- not found	n.a.	- No dbSNP mapping(s)	- score 0.466
							- neutral
c.611 + 3366C > T	intronic	n.a.	n.a.	n.a.	n.a.	-n.a.	n.a.
rs61733438	Asn321Ser	- tolerated	- tolerated	- disease causing	- aas changed	- tolerated	- score 0.278
				- aas changed		- score -0.92	- neutral
				- heterozygous in TGP	- disease associated variation		
				- protein features (might be) affected			
				- splice site changes	(probability – 0.848)		
rs2148328	Ala399Gly	- tolerated	- unknown	- aas changed	- aas changed	- No dbSNP mapping(s)	- score 0.540
				- protein features (might be) affected			- “Actionable Hypotheses”^$^

^$^) Loss of relative solvent accessibility (P = 0.0071); Gain of loop (P = 0.0166); Loss of helix (P = 0.0376); Loss of solvent accessibility (P = 0.0442); aas = amino acid sequence, TGP = 1000 Genome Project.

## Discussion

The development of the distinct cell lines of the pituitary gland is directed by nuclear mediators of cell type commitment, including the BMP pathway and a number of transcription factors (reviewed in [2]). The role of BMP2, BMP4 and BMP7 as signalling peptides in the programming of pituitary development makes them plausible candidates for pituitary disorders including congenital insufficiency as well as pituitary adenomas. In our study we systematically screened for genetic variation in these genes in a group of patients with CPHD.

Inhibition of *Bmp2/Bmp4* in mice causes loss of the Pit-1 lineage and gonadotropes but not of POMC-expressing cells [8]. In detail, BMP2 is essential for the expression of ventral markers such as the insulin gene enhancer protein ISL-1 and human glycoprotein hormone α-subunit gene and necessary for terminal differentiation of pituitary cell types [8,27]. We could identify two known SNPs, rs2273073 and rs235768, in two different patients. To predict the potential impact of an identified variant on protein structure and function we used several online tools and databases [20-25]. However, we are aware of limitations of these tools and used comparative considerations and degree of conservation of amino acid residues do not provide functional evidence. The p.Ser37Ala substitution caused by rs2273073 is assumed to be tolerated according to SIFT and PolyPhen whereas it is predicted to be disease associated by Mutation taster and SNP&GO. The carrier of this variant in our study did not present any further phenotype other than CPHD. There is evidence in the literature that variation at rs2273073 affects bone mineral density [28] but we do not possess any clinical data on this phenotype in our study. Additionally, we identified rs235768 predicting the p.Arg190Ser exchange in one subject in our cohort of CPHD patients. An association of p.Arg190Ser substitution with the development of childhood IgA nephropathy has been described [29], the functional relevance cannot be predicted explicit based on SIFT and PolyPhen database search, it described as neutral or tolerated by MutPred and FATHMM but potentially disease associated by SNP&GO. Further functional studies are required to elucidate detailed effects of this variant.

In accordance with previous studies [27] our phylogenetic analyses of the *BMP4* gene revealed a highly conserved sequence of the *BMP4* region which would suggest a potential functional relevance of variation at this locus. We identified a novel variant resulting in a c.899G > C substitution predicting a missense mutation within the protein sequence (p.Arg300Pro). Bioinformatic prediction tools provide substantial evidence to functional consequences and the fact that we have not found a second heterozygous or homozygous c.899G > C carrier in a set of 1046 healthy subjects and the high conservation at this locus furthermore support a potential association with the phenotype. An X-ray of the patient at the age of 17 presents skeletal abnormalities described as vertebral platyspondylia, sclerosis of the metaphyses and a short metacarpalia IV, which would be in line with the diagnosis of spondyloepiphyseal dysplasia tarda. Since *BMP4* is known to increase osteoblast differentiation the affection of the skeletal system would be consistent with a functional relevance of the newly identified c.899G > C substitution. Furthermore, fibrodysplasia ossificans progressiva is characterized by an overexpression of *BMP4* in lymphocytes [30], so detailed functional analyses are required to assess effects on *BMP4* expression and interaction with BMPR1A receptor pathway. A detailed family history or genetic material of the patient’s family are unfortunately not available which is a clear limitation of the study. According to the self reported family history all other relatives do not show any affection of the pituitary function. However, it is of note that there is a substantial variability in the clinical presentation of patients with combined pituitary hormone deficiency even if the same gene is affected and even in subjects with identical mutations. Intra-familial penetrance can range from high to incomplete and it is not possible to draw direct conclusions form the clinical manifestation to the potential genotype. This indicates the remarkable influence of the genetic background, incomplete penetrance, highly variable expressivity, environmental factors and possibly stochastic events. Also co-occuring mutations in interacting genes have to be taken into account.

Additionally to this new variation we found the SNP rs17563 in the coding region of *BMP4*. This variation has been suggested to be involved in the development of otosclerosis [31]. According to the high prevalence in healthy subjects an association with pituitary disorders is rather unlikely.

*BMP7,* also called Osteogenic Protein 1 has an important function during the embryonic development of the eye, brain and ear [17,18]. In mice, Bmp7 is responsible for the expression of *Pax6* and *Sox2*[32] that are both known to be involved in the development of the pituitary gland [3]. We have identified rs61733438, resulting in p.Asn321Ser substitution. So far, rs61733438 has been described in patients with several eye defects [33]. The male patient identified in our CPHD cohort who is carrier of the heterozygous rs61733438 variant has an ectopic neurohypophysis but no other specific symptoms. Furthermore, there is no family history of CPHD.

Taken together, we identified several genetic variants in *BMP2, BMP4* and *BMP7* in a group of patients with CPHD. However, genotyping of further patients and mainly functional analyses are required to clarify the exact role in pituitary insufficiency. Clear limitation of our study is the missing genetic information for family members. These data would significantly support phenotype-genotype associations and would strengthen potential functional relevance of the identified variants. Furthermore, the group of CPHD patients included in our study presents a heterogeneous phenotype and most likely also diverse genetic source. We are also aware that by including only a few genes the data remain inconclusive. However, we believe that even by extending the list of studied genes by further candidates there would be no guarantee that further players will be identified. Thus, a systematic approach including whole genome/exome sequencing strategies would be desirable here.

## Conclusions

Our study provides a systematic analysis of *BMP* genes in patients with CPHD. We identified novel variants in *BMP2, BMP4* and *BMP7*. Of particular interest is a novel variant in *BMP4* (p.Arg300Pro) found in one patient with skeletal malformation in addition to CPHD. Further functional characterization of the newly identified variant is desirable not only to ultimately pinpoint their biological and clinical consequences but also to better understand the role of bone morphogenetic proteins in the pathophysiology of congenital combined pituitary insufficiency.

## Competing interests

The authors declare that they have no competing interests.

## Authors’ contributions

JB and SM sequenced the genes, analyzed the results, contributed to discussion and drafted the manuscript. JK and RP participated in the design of the study, provided samples and contributed to discussion. KW carried out the evolutionary analyses. DF and MS contributed to the study design and discussion of the results. PK and DS analyzed the results, contributed to discussion and edited the manuscript. AT designed the study, provided samples and contributed to discussion and manuscript writing. All authors read and approved the final manuscript.

## Pre-publication history

The pre-publication history for this paper can be accessed here:

http://www.biomedcentral.com/1472-6823/13/56/prepub

## Supplementary Material

Additional file 1 PCR conditions. Click here for file

Additional file 2 Primers. Click here for file
